# Morphology and Growth of *Arthrospira platensis* during Cultivation in a Flat-Type Bioreactor

**DOI:** 10.3390/life11060536

**Published:** 2021-06-09

**Authors:** Conrad H. G. Jung, Steffen Braune, Peter Waldeck, Jan-Heiner Küpper, Ingolf Petrick, Friedrich Jung

**Affiliations:** 1 Carbon Biotech Social Enterprise AG, 01968 Senftenberg, Germany; c.jung@carbonbiotech.eu (C.H.G.J.); Jan-Heiner.Kuepper@b-tu.de (J.-H.K.); 2 Institute of Biotechnology, Molecular Cell Biology, Brandenburg University of Technology Cottbus-Senftenberg, 01968 Senftenberg, Germany; steffen.braune@b-tu.de; 3 Institute of Materials Chemistry, Thermodynamics, Brandenburg University of Technology Cottbus-Senftenberg, 01968 Senftenberg, Germany; peter.waldeck@b-tu.de (P.W.); Ingolf.Petrick@b-tu.de (I.P.)

**Keywords:** *Arthrospira platensis*, Spirulina, life cycle, morphology, fragmentation, fission

## Abstract

*Arthrospira platensis* (AP) is a cyanobacterium with a high economic value and is nowadays one of the most important industrially cultivated microalgae. Knowledge of its growth is essential for the understanding of its physiology and yield. The growth of AP biomass occurs through two mechanisms: (1) propagation by fragmentation of trichomes, and (2) the trichomes are extended by binary fission until they reach their mature status. These phases are visualized by live cell light and laser scanning microscopy, demonstrating the different phases of AP growth.

## 1. Introduction

*Arthrospira platensis* (AP) is a species of cyanobacterial phylum. Cyanobacteria typically carry out oxygenic photosynthesis with water as an electron donor and use carbon dioxide as a carbon source. This cyanobacterium (often called blue-green alga) grows as filamentous, helicoidal trichomes, performs oxygenic photosynthesis and reproduces by binary fission [1]. AP has a long history of use as food and gained considerable popularity in the human health food industry due to its therapeutic properties, including antioxidant, anti-inflammatory, immune-modulatory and anticancer activities [2,3,4]. In many countries of Asia, it is used as a protein supplement, as human health food and as feed for poultry and aquaculture. Nowadays, the successful commercial exploitation of AP due to its high nutritional value, chemical composition and the safety of the biomass has made it one of the most important industrially cultivated microalgae [5]. 

**Figure 1:**
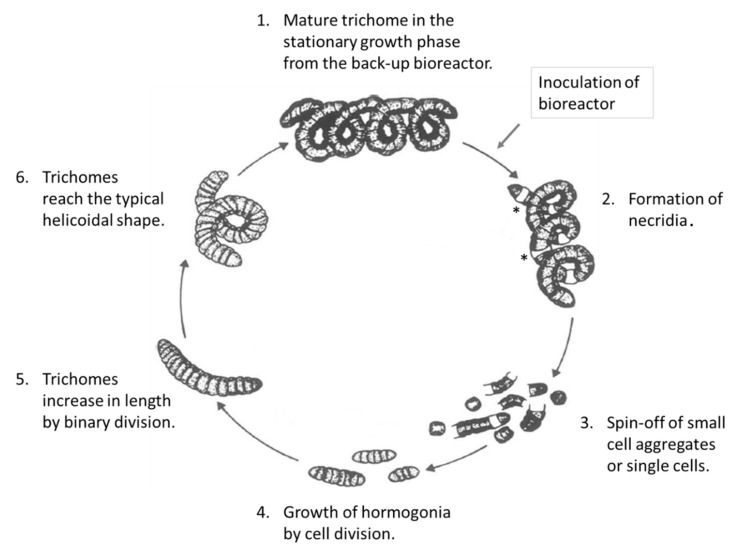
Life cycle of *Arthrospira platensis* (modified according to [9]). The asterisk (*) indicates necridia.

Knowledge of its physiology is essential for understanding its growth status. The main factors for the growth of AP are light and CO_2_ or HCO_3_¯, respectively [6,7,8]. The life cycle of AP (see Figure 1) was first described by Ciferri [9]. Here, we investigated the morphology of AP (strain: SAG21.99, Göttingen, Germany) during growth in a bioreactor from the 1st day up to the 7th day via different live cell imaging techniques.

## 2. Materials and Methods

**Figure 2:**
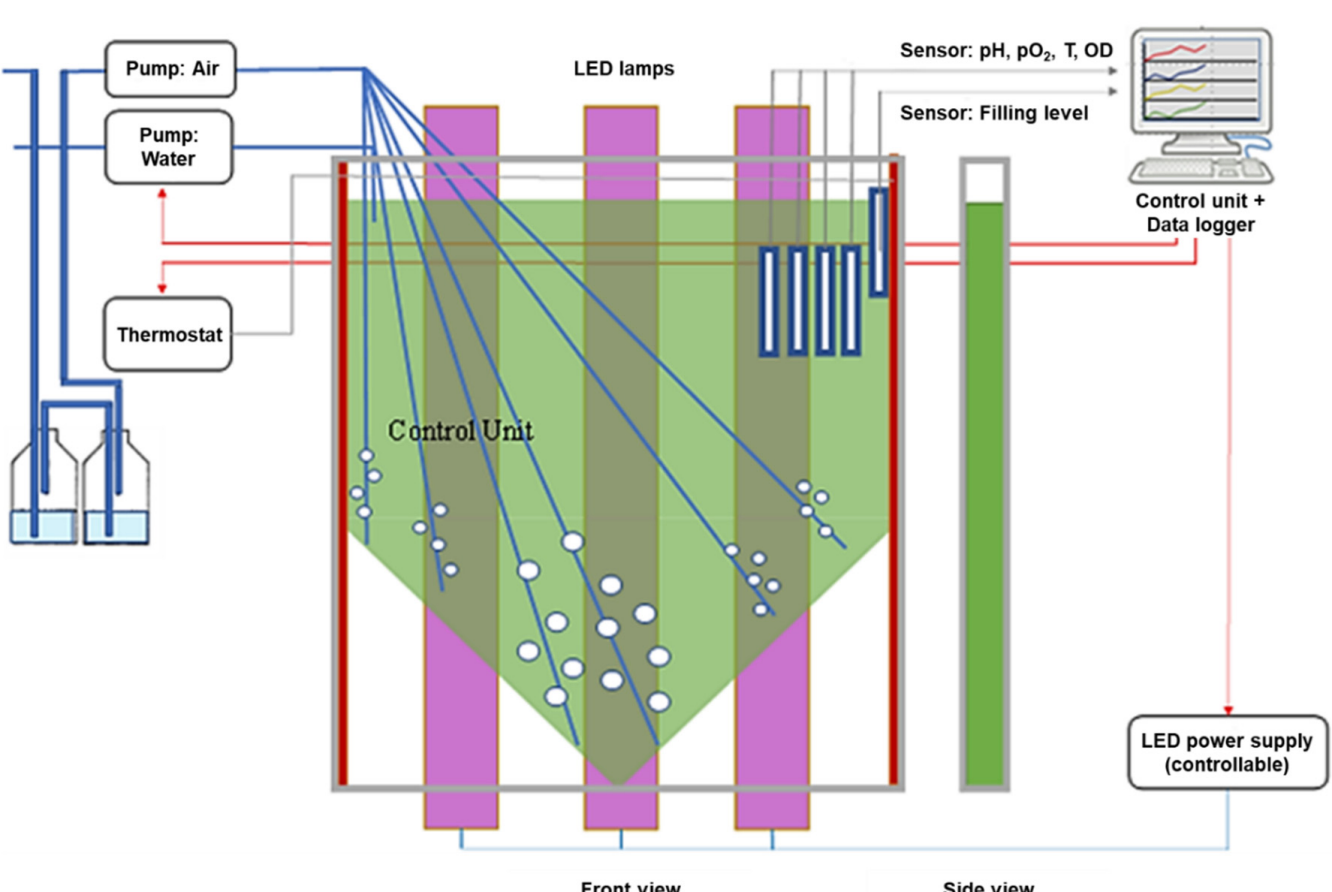
Sketch of the bioreactor for the production of *Arthrospira platensis (adapted* from [11]).

AP used for cultivation was obtained from the “The Culture Collection of Algae at Goettingen University” (strain: SAG21.99). *AP* were cultured in a flat-type (2 cm) vertical transparent bioreactor consisting of a flexible polyethylene (PE, food safe grade) sleeve with a 1.0 L working volume. The PE sleeve was pressed between two adaptable polymethyl-methacrylate plates (see the green reactor in Figure 2). For the experiments, Zarrouk medium was used [10]. The growth medium was initially sterilized at 121 °C in a HV-50 autoclave (SYSMEX VX-95, Sysmex, Norderstedt, Germany) for 15 min. The bioreactor was inoculated with AP cells (0.19 g/L) from a light-limited—because of the shading of the high cell density—back-up bioreactor in which the AP had already reached the stationary growth phase. At this stage of development, AP cells were transferred into a bioreactor filled with Zarrouk medium, aerated with air supplemented with 2% CO_2_ and illuminated with a blue-red LED lamp (AP673L, Valoya, Helsinki, Finland) set to 250 µmol/(m^2^ * s) at the bioreactor surface for up to seven days. Stirring of the culture suspension was carried out using six tubes so that sufficient mixing of the culture medium was achieved. Air was pumped through a membrane filter (Millipore; 0.45 µm pore size, 10 cm diameter) and moistened by passaging it through distilled water, with a flow rate of 200 L/h, respectively. Aeration was adjusted using area flow meters. The appropriate air volume flow was measured in pretests so that the pH value of the growth medium was maintained between pH 9 and pH 10.0 for the duration of the experiment. The filling level was kept constant to compensate for evaporation losses. The temperature in the bioreactor was maintained at 25 °C. Light intensity was measured using a LI-250 light meter with a LI-190SA pyranometer sensor (LI-COR, Inc., Lincoln, NE, USA). The optical density (Thermofisher, Genesys 100 Bio, Waltham, MA, USA), temperature (PT1000, Wernberg, Germany), pH values (EGA 133, Sensortechnik Meinsberg, Meinsberg, Germany) and oxygen concentration (FDA120, Hamilton, Bonaduz, Switzerland) of the culture medium were monitored during the cultivation time continuously. A sketch of the bioreactor is shown in Figure 2 (for details, see [11]).

**Figure 3:**
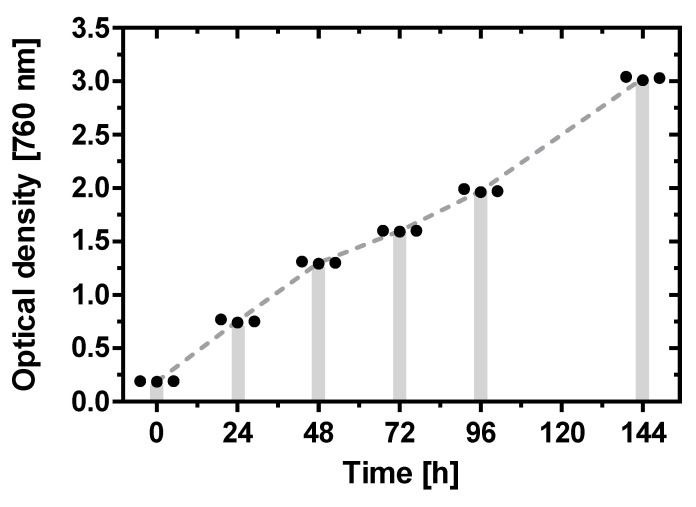
Growth curve for *Arthrospira platensis* in the described bioreactor over seven days. For each time point, three individual measurements of the optical density (at 760 nm) of the cell solution are given as staggered black point symbols. The dashed lines indicate a linear trend between the mean values at each time point. The gray vertical bars indicate the measurement time points.

On each experimental day, samples were taken from the bioreactor and were examined via bright field and phase contrast microscopy (Axio Scope, Zeiss Microimaging GmbH, Jena, Germany; BZ-X810, Keyence, Japan). Samples were further studied by laser scanning microscopy (Axio Observer.Z1/7, Zeiss Microimaging GmbH, Jena Germany). Geometry measurements of the trichomes were carried out with ImageJ (National Institute of Health, Bethesda, MD, USA) [12]. Figure 3 shows the growth curve of AP over seven days.

## 3. Results and Discussion

A flat panel-type bioreactor with a depth of 2 cm (minimizing the cell-induced shading) was used to avoid self-shading and to achieve many hormogonia. To avoid phototoxicity, sufficient nutrient concentrations are needed. This was attained by full Zarrouk medium and an additional 2% CO_2_ in the aerating gas flow. The high aeration rate of 200 L/h guaranteed a homogeneous culture and light availability.

Those light-limited AP were mature (see 1. in Figure 1) and started to divide as soon as the light intensity increased. Basically, the growth of AP biomass occurs through two mechanisms: (1) propagation occurs by fragmentation of trichomes, and (2) the trichomes are extended by binary fission [13,14] until they reach their mature status.

**Figure 4:**
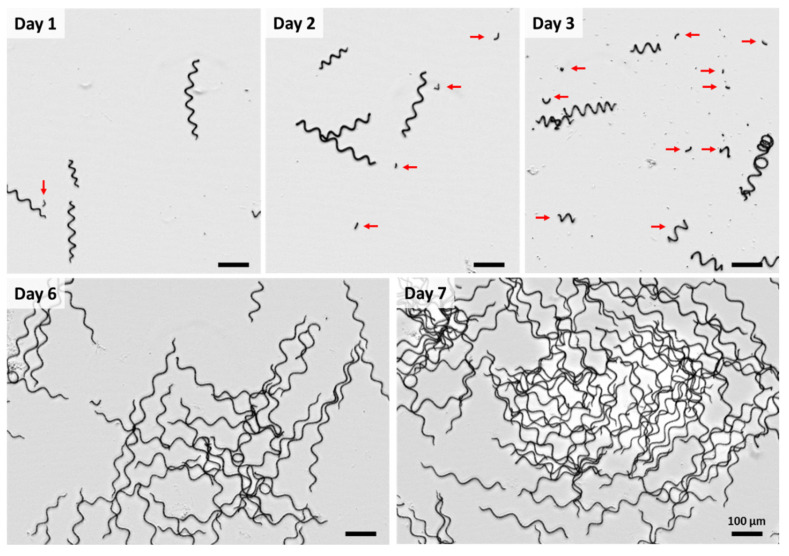
Representative overview images of the morphological characteristics of *Arthrospira platensis* (strain: SAG21.99) during the seven days’ cultivation. Red arrows indicate short trichome fragments of *Arthrospira platensis*. Bright field microscopy, Axio Scope, Zeiss Microimaging GmbH.

**Figure 5:**
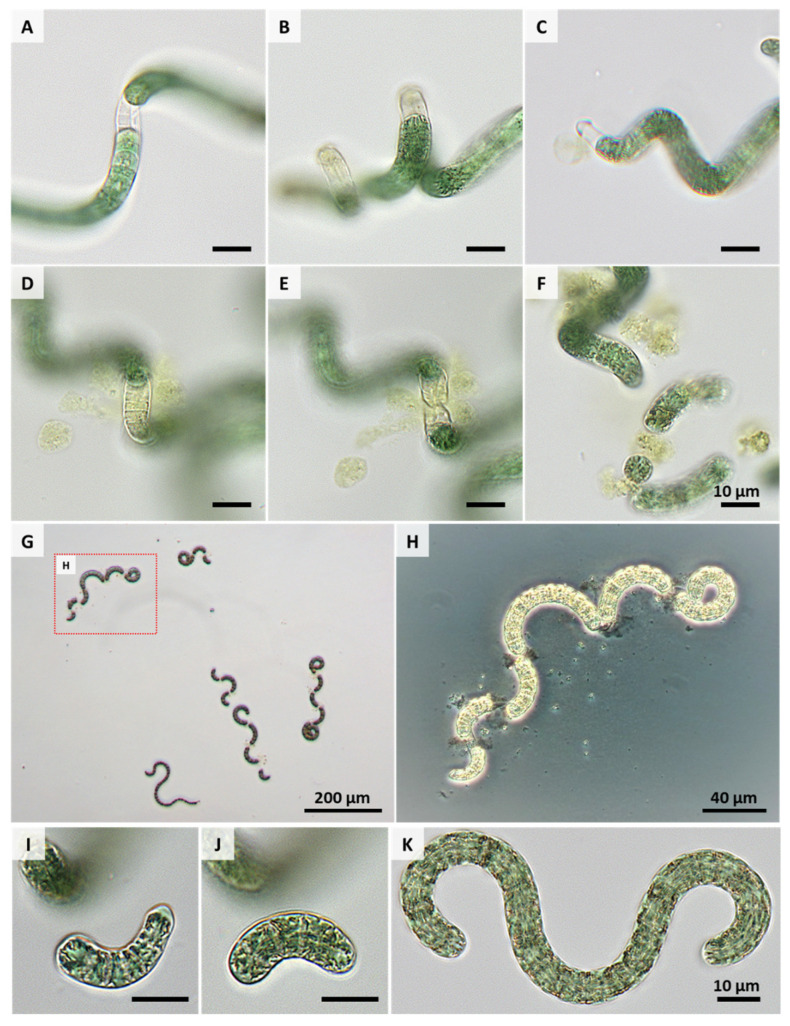
Representative images of light-induced necridia formation (**A**,**B**: intermediate cells, **C**: terminal cell), (**D**–**H**) fragmentation and (**I**–**K**) hormogonia/trichomes in light-limited AP (strain: SAG21.99). (**G**–**H**) Simultaneous multiple fragmentation of AP at very high illumination (2000 µmol/(m^2^ * s)) and sufficient HCO_3_¯. (**A**–**F**,**I**–**K**) Bright field microscopy, BZ-X810, Keyence, Japan, (**H**) phase contrast microscopy, Axio Scope, Zeiss Microimaging GmbH, Germany.

**Figure 6:**
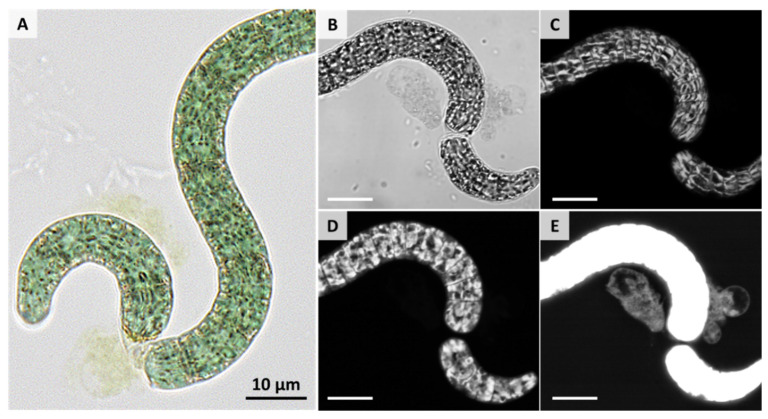
Representative images of the fragmentation and release of the cytoplasm content into the extra cellular space. (**A**) bright field microscopy at a 40-fold primary magnification (BZ-X810, Keyence, Japan). (**B**–**E**) Label-free laser scanning microscopy of unfixed AP cells. Samples were exited at a 555 nm wavelength. Emissions were detected between 650 nm and 700 nm. (**B**) represents a transmitted mode image. (**C**,**D**) show images from different z-levels of the same x-y position. (**E**) shows an image with enhanced fluorescence intensities to show the fluorescence properties of the former cytoplasmatic content. Scale bar represents 10 µm. Images were taken with an Axio Observer.Z1/7, Zeiss Microimaging GmbH, Germany.

Figure 4 shows the development of AP over the cultivation period. At the first day after the formation of necridia, only very few short trichome fragments of AP cells (see the arrow in the figure for day 1) were found. Necridia are specialized cells within the filament formed during the propagation of AP, which is accompanied by cell lysis and the formation of debris (see Figure 5A–H and Figure 6A–E). Due to the fragmentation of the trichome at the necridia, short chains of cells (of up to 10 cells), the hormogonia, originate (see red arrows in Figure 4 and Figure 5I–K). These cell aggregates move away from the parental filament and give rise to a new trichome. The cells in the hormogonium lose the attached portions of the necridia with a substantial cell debris formation (see Figure 5A–H). Laser scanning microscopy revealed (Figure 6E) that the former cytoplasmatic content retained its fluorescence properties in the extracellular space. However, visualization was possible only when the sample exhibited a substantially stronger laser excitation, compared to the other images (compare Figure 6B–E).

The number of hormogonia increased significantly up to day 3 (see red arrows in Figure 4). At the same time, the divided cells started to grow immediately due to the abundant light and HCO_3_¯ availability, the main factors inducing AP to grow [15]. During this process, the trichomes increased in length and reached the typical helicoidal shape. This trichome elongation occurs through multiple intercalary cell division by binary fission at right angles to the long axis of the trichome [16].

Simultaneous multiple fragmentations of trichomes were observed only when AP from the stationary growth phase were illuminated with a high photon flux density and sufficient HCO_3_¯. Such an event is shown in Figure 5G,H. Under such conditions, necridia are formed and the highest growth rates occur. Ma et al. have confirmed earlier studies showing that upon exposure of AP to photosynthetic active radiation (e.g., solar, ultraviolet), reactive oxygen species are generated [17]. These can oxidize lipids of the cell membrane or the sheath, which can lead to the breakage of the spiral structure. It seems conceivable that the conditions applied in this study might induce similar processes, leading to the formation of necridia and the release of the intracellular content. However, shear stresses or cell-cell contact processes have also been discussed as reasons for AP breakage in bioreactors and should be considered as well.

After seven days, the trichomes had lengths between 100 µm and 500 µm (only very few short spin-offs were still visible at that time) with an outer helix diameter between 20 µm and 60 µm. This corresponds well with former data by Ciferri for AP [9]. However, it is worth noting that the helix geometry of AP strains can be influenced by certain environmental variables such as light, temperature, pH, salinity and nutrient availability [18,19,20], so that different morphologies can also result.

The AP diameters decreased from 7.5 ± 2.9 µm on day 1, to 6.5 ± 1.7 µm on day 2, to 5.75 ± 1.46 µm on day 3, to 5.25 ± 1.26 µm on day 6, and to 6.75 ± 1.7 µm on day 7. Thus, after seven days, the cells almost reached the diameters of those from the initial back-up bioreactor culture.

## 4. Conclusions

Live cell microscopical observations over the cultivation time demonstrated the growth process of AP in a bioreactor setup. The division of mature spirals is a physiological reaction—not a pathological sign—as soon as they are exposed to a high photon flux density and sufficient nutrients. Beyond the classically applied bright field and phase contrast light microscopy, high-resolution laser scanning microscopy can also be utilized for studying nonfixed AP samples. The image-based techniques can be used for fundamental scientific studies. But also—in addition to recording the optical density—to find the adequate time for harvesting large mature AP spirals with size-adapted meshes and to lead back the filtrate—small cell aggregates or single cells—to the next growth cycle.
